# The Impact of Changes in Health and Social Care on Enteral Feeding in the Community

**DOI:** 10.3390/nu4111709

**Published:** 2012-11-13

**Authors:** Omorogieva Ojo

**Affiliations:** Department of Acute and Continuing Care, School of Health and Social Care, University of Greenwich, Avery Hill Campus, London, SE9 2UG, UK; Email: o.ojo@greenwich.ac.uk; Tel.: +44-20-8331-8626; Fax: +44-20-8331-8060

**Keywords:** enteral feeding, collaboration, community, health, social care, challenges, opportunities

## Abstract

This paper examines the impact of the changes to health and social care on enteral feeding in the community, outlines implications for practice and offers recommendations to ameliorate the challenges. It is now clear that there have been significant changes especially in the last 10 years in health and social care provisions in the UK with an overarching effect on enteral nutrition in the community. Advances in technology, increasing demand and treatment costs, the need for improvement in quality, economic challenges, market forces, political influences and more choices for patients are some of the factors driving the change. Government’s vision of a modern system of health and social care is based on initiatives such as clinically led commissioning, establishment of Monitor, shifting care from acute hospitals to community settings, integrating health and social care provisions, Quality, Innovation, Productivity and Prevention (QIPP) program and the concept of “Big Society”. These strategies which are encapsulated in various guidelines, policies and legislation, including the health and social care Act, 2012 are clarified. The future challenges and opportunities brought on by these changes for healthcare professionals and patients who access enteral nutrition in the community are discussed and recommendations to improve practice are outlined.

## 1. Introduction

There have been changes in the UK National Health Service (NHS) since its inception in 1948 although the changes have been profound in the last 10 years [1]. The developments in health and social care provisions have significant impact on enteral feeding in the community and appear to be driven mainly by resource allocation, market forces, political influences, advances in technology and the need for better patient treatment. Over the period, there has been a move away from a system that is primarily driven by national targets to that where standards are the main drivers for continuous improvement in quality [2].

The individuals suffering from cardiovascular diseases, cancer and diabetes which are among the leading cause of death globally may require enteral feeding due to related complications such as stroke [3]. These people are now better diagnosed and managed, and are in fact living much longer due to advances in technology and developments in scientific knowledge [4]. For example, the number of patients with head and neck cancer and cerebral vascular accident (CVA) who may require enteral nutrition due to dysphagia is now on the increase [5,6]. In addition, people’s expectations about how their feeding tubes are managed are changing with respect to making decisions, choices and independence [7].

The dwindling healthcare resources has placed a burden on the NHS in the allocation of scarce resources between acute and community care settings [8]. The government in recognition of the trend including the global economic recession has come up with various policy documents and initiatives which have effect on enteral feeding. The drive towards primary care led NHS is one of the strategies being adopted by government which is aimed at addressing some of the health and social care challenges. This means that new ways of working have to be developed including the need for multidisciplinary collaboration and cooperation in the care of patients on enteral feeding in the community [9]. The target of government is to create greater flexibility in service provision, better accessibility and minimal disruption to daily lives [10]. However, these changes have come with challenges and opportunities across the broad spectrum of health and social care provisions especially with respect to Home Enteral Nutrition (HEN) service in terms of professional roles and responsibilities, hierarchy, recruitment and retention. These changes will no doubt have impact on health and social care professionals, providers and patients who access enteral feeding in the community. 

Therefore, this article is aimed at providing an overview of the potential challenges and prospects that the changes in health and social care bring in relation to enteral feeding in the community. 

One of the government’s plans is to integrate health and social care provisions including partnership working between health and social care professionals caring for people with long term conditions such as CVA patients on enteral feeding in the community [7,11]. While some people may tolerate oral dietary intake, many are unable to meet their nutritional requirements through oral intake alone due to factors such as dysphagia. Thus, it is possible to have people with long term conditions in the community on oral dietary intake alone, a combination of oral dietary intake and enteral feed, and those on enteral feed only. Many patients with enteral feeding tube in–situ often have complex needs and live in the community. The management of these patients involves the use of different approaches and often requires the expertise and collaboration between different health professionals in order to ensure good clinical outcomes. Collaboration and cooperation are essential parts of the roles of nurses and other healthcare professionals in order to achieve a common goal [12,13]. However, there are factors which hinder and facilitate collaboration in a multi-disciplinary team such as the HEN team, including organizational and individual factors [14,15].

## 2. Present State of Enteral Feeding in the Community

**Figure 1:**
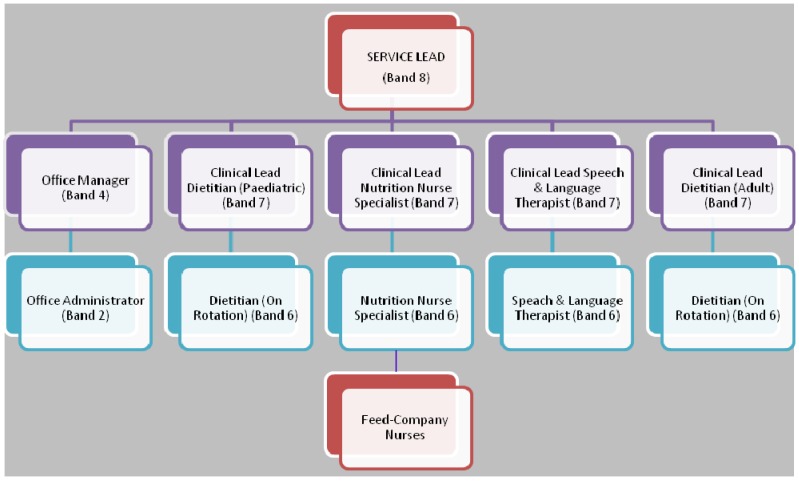
A model of the Home Enteral Nutrition (HEN) service provided by Lewisham Healthcare NHS Trust.

With respect to nutrition support services that are based in the community, traditionally, these include a General practitioner (GP), the community nurse and sometimes a dietitian [16]. However, the number of patients on Home Enteral Tube Feeding (HETF) in the UK is on the increase partly due to better diagnosis of medical conditions, improved technology and increased longevity in patients with long term conditions [5]. The response of government to this change has been to establish Enteral Nutrition Teams that include the dietitian (hospital or community based) and feed-company nurses. This approach has not fully addressed the complex needs of these patients. On the other hand, the HEN services which have Nutrition nurse specialist (NNS), Dietitian, Speech & language therapist and Feed-company nurses are few in the UK and their establishment and effectiveness come with enormous challenges. A model of the HEN service provided by Lewisham Healthcare NHS trust is depicted in Figure 1.

## 3. Main Factors Driving Changes in Health and Social Care in Relation to Enteral Feeding in the Community

Some of the factors driving the change in health and social care include; the rising service demand and treatment costs, need for continuing improvement in service delivery and the state of public finance [18]. For example, the cost of medicines is growing by over £600 million per year in the UK [18].

The coalition government has outlined their vision including the need for deficit reduction and spending review [7]. In order to achieve up to £20 billion efficiency gains by 2015, fundamental changes in the whole health system would be required [19]. Approaches such as reduction in the overall number of staff employed in the NHS and the workforce for Quality, Innovation, Productivity and Prevention (QIPP) program which supports NHS staff to have skills needed to deliver the highest standards of patient care are being pursued by government [11,19].

In addition, government has enacted the health and social care Act involving the development of new commissioning structures for NHS alongside local authorities [20]. While the long term effects of these changes on enteral feeding in the community will be far reaching in terms of training of specialists in this area of practice, the impact on patients will be profound.

The main external drivers of future service requirements and changes to health and social care include medical advances (better diagnosis and treatment of diseases), information technology (improved discharge planning), innovative services, evidence based practice and patients knowledge of their conditions and demand for choices [1,21]. Others include the changing demographics and nature of disease. For example, by 2031, the number of those over 75 years old in the British population will increase from 4.7 million to 8.2 million, representing a difference of 74.5% [4]. In addition, the 4.7 million over 75 years old represent about 7.5% of the 62.3 million UK population [22]. This older group uses a disproportionate amount of NHS resources; the average over 85 years old is 14 times more likely to be admitted to hospital for medical reasons than the average 15–39 year old [4]. In addition, the choices people make today including smoking, excessive drinking of alcohol, poor diet and lack of physical activities are risk factors for a range of diseases such as cardiovascular diseases, cancer and diabetes [3]. Certain cancers and stroke may require enteral feeding [6]. The role of the HEN service will be crucial in meeting these challenges.

On another note, the number of care homes with nurses is much smaller than those without nurses. For example, in 2011, the numbers of care homes with nurses were 4068 compared to the 13,475 care homes without nurses [20]. The implication is that patients with enteral feeding tube in care homes without nurses are more likely to require specialist intervention from nutrition nurses compared with those with nurses. Social care provisions are also changing with increasing demand for services and allowing more people to live at home for longer [20].

The government is also providing more choices so that patients can make decisions over the care they receive. Over the years, NHS bed capacity for people with learning disabilities has been replaced by options in voluntary and private care homes or in group or individual accommodations supported by social care. Similarly, longer term for older people in care homes and community settings is on the increase [20]. For learning disability patients on HETF in these settings, more responsibility will be required for training and retraining of carers in order to ensure good clinical outcomes.

The fall in the number of hospital beds from 480,000 when NHS was set up in 1948 to 190,000 in 1998 [1], is another factor driving the change. This difference of 290,000 beds will no doubt require more support from community NNS for HETF patients who are discharged home early. 

## 4. What Are the Changes and the Implications for Enteral Feeding Practice in the Community?

The changes in health and social care have come in the form of polices, programs, guidelines, medical alerts and legislation. 

They include: 

■ Shifting of care from hospital to community.

The shifting of care from acute to community settings will also require changes in the roles and relationships of service users on enteral feed, commissioners and providers in order to achieve the desired results [10]. According to NHS Institute for Innovation and Improvement [10], this would involve:

- Integrating primary and secondary services (e.g., adding NNS to primary care teams, integrating health and social care). Integrating health and social care services is a useful strategy of managing changing demographics, enhancing the quality of patient care, improving innovation and development, and saving costs [23,24]. For example, the integration of Lewisham hospital NHS trust with Lewisham community health services which led to the formation of Lewisham healthcare NHS trust offers patients a continuous pathway of care through primary, community and hospital care [25]. This integration is useful for enteral feeding in the community in terms of managing the patients on HETF. In addition, enteral nutrition practitioners in the community can now effectively provide training for staff in the hospital and ensure better discharge arrangement for patients where there is no NNS in hospital settings and vice versa;
- changing location (e.g., providing care at home, intermediate care);
- substituting skills (e.g., service users for professionals, substituting nurses for other professionals and adding the skills of NNS);
- simplifying pathways;
- The Transforming Community Services’ (TCS) program includes rehabilitation of patients with long term neurological and other long term conditions who may be on HETF [24]. The TCS programs have involved mergers of Community Health NHS trusts and integration of acute with community services [20]. One of the strategies of bringing care closer home is the delivery of specialist care in community settings with the help of multidisciplinary teams such as the HEN service [9]. This will no doubt make the HEN services better accessible and convenient for patients in the community.

In addition, the intermediate care service was developed to ensure a smooth transition from hospital to home and prevent avoidable admissions to acute care settings [1]. In order to ensure effectiveness of the intermediate care services, the department of health suggested that multi- disciplinary community based teams could coordinate the activities across the sectors (hospital, outpatient, and community) in order to reduce the blurring of boundaries. 

The care closer to home scheme ensures that health and social care professionals work as a multidisciplinary team with the development of new roles and skills [1]. This would involve manpower and training implications in terms of training of NNS on how to care for vulnerable adults and for social workers on how to recognize and to report enteral feeding complications [1]. Rehabilitation skills for NNS will be required with more elderly people on HETF being discharged to the community. More inputs by healthcare professionals working in HEN service are now required due to the reduced length of time people spend in hospitals [20]. 

■ Health and Social Care Act, 2012.

Some of the key legislative changes in the health and social care Act include the establishment of the NHS Commissioning Board and the new clinical commissioning groups (CCGs) which will now directly commission services [18,19]. In addition, patients will be free to choose services and have greater voice, providers will be able to transform services and Monitor will be established to regulate providers of NHS services [18]. While the purpose of the reforms is to extend choice and competition, patients and healthcare professionals working in the area of enteral feeding are however concerned whether this Act will end the pledge to deliver universal health services that are largely free at the point of use [26]. 

Although the Strategic Health Authorities (SHAs) have been merged to form four clusters in England and plans are in place to have the primary care trusts (PCTs) merge to 51 clusters, it is hoped that the eventual abolition of the PCTs and SHAs in 2013 will improve integration between NHS and local authority services and enhance the management of patients on enteral feeding in the community [19,26,27]. The plan is to replace SHAs and PCTs with the NHS commissioning Board and CCG that will be responsible for commissioning projects [26]. It is hoped that these changes will improve the quality of care provided to HETF patients while at the same time saving costs.

Apart from the concern about privatization of the NHS by the new legislation there are issues about the role of Monitors. According to Ham [26], the original duty of Monitor to “promote competition” has now been removed. On the other hand, Davies [28], argued that Monitors should be there to protect the interest of the patients and not primarily to promote competition. 

■ The National Institute for Health and Clinical Excellence (NICE) Guidelines. 

The NICE guideline on infection prevention and control of healthcare associated infections (HCAIs) in primary and community care [29], is an update of the 2003 guideline [30]. The new guideline sets out recommendations for healthcare professionals, patients and carers on various aspects of managing enteral feeding in the community. The risks presented by HCAIs can be reduced if there is higher degree of compliance to the NICE recommendations by all stake holders involved in enteral feeding in the community.

NICE has also carried out a scoping exercise on quality standards on nutrition support in adults (18 years and older) [31]. The standards covered adults who are receiving oral nutrition support, enteral or parenteral nutrition in all health and social care settings where these provisions are established. Areas of care that were considered by the Topic Expert Group amongst others included:

- Screening for malnutrition and risk of malnutrition;
- Nutritional assessment;
- Recording of nutritional information (e.g., nutritional status, goals, care plan and prescription);
- Nutritional prescription of total intake;
- Principles of treatment of underlying causes;
- Quality of documentation and audit;
- Continuity and consistency of care.

Following this exercise, NICE has produced the draft quality standard for nutrition support in adults which is aimed at improving the quality of care for people requiring enteral feeding [32]. This standard requires that people at risk of malnutrition are identified by all care services and that nutritional support is provided to all the people who need it [32]. 

Other strategies being adopted by the government is the SHAs and Department of Health developed Tripartite Formal Agreements (TFAs) which outline the challenges that NHS trusts face and what actions should be taken to become clinically and financially sustainable including driving the process leading to full Foundation trust [19]. The Government has a strategy to ensure that all NHS providers become Foundation trusts by 2014 [26]. However, there are concerns that as hospitals are affected by spending reviews they may be compelled to treat wealthy foreigners rather than HETF patients in order to raise money [28].

Also, service re-design such as the hyper-acute stroke units in London have helped to improve services for stroke patients and with implications for HETF patients [19]. The streamlining of services, reviews and re-banding of staff have significant effect on HEN provision. In addition, HEN services are being requested to demonstrate their effectiveness by reporting on activities, developing/reviewing care pathways and clinical outcomes. Another fundamental impact of the current changes in health and social care in the community is the issue of the procurement and supply of oral nutrition supplements – which are currently being prescribed in the community. The usage of these supplements is being reviewed in relation to spending cuts.

The government’s concept of the “Big Society” in relation to health and social care entails giving people the power to support one another in order to address the challenges they face and maintain their independence [11]. This may be useful in assisting HETF patients with good manual dexterity to manage their feed and feeding tubes independently. 

## 5. Challenges and Opportunities Resulting from Changes in Health and Social Care

The shifting of care from secondary to primary care settings presents with challenges and prospects. The issue of resources for staff training including continuing professional development (CPD) for both allied healthcare professionals (AHPs) and nutrition nurses and potential to undermine relationship between secondary and primary care are some of the potential challenges [9]. Others include maintaining the quality of care and ensuring patient safety, extending patient pathway of care and meeting the demands of integrated services [9]. In order to alleviate some of these challenges, it may be essential to have adequate workforce planning, effective clinical governance set up and adequate service level agreements between commissioners and providers [9].

Transforming the health and social services including integrated services and multidisciplinary team (MDT) working may create several opportunities [15]. The level of skill mix and the depth of knowledge within the HEN team provide advantages and opportunities for the team and the patients’ it serves [33]. To fully utilize the skill mix and expertise in the team it may be necessary to extend the scope of practice of members of the HEN team. A checklist of what is needed to implement this way of working includes:

♦ Finding out about professional indemnity/professional code of conduct for the different disciplines (NNS, Dietitian, Speech and Language Therapist–SLT);
♦ Agreeing what tasks can safely be shared between disciplines, e.g., application of MDT assessment tool, weighing patients and checking enteral feeding tubes;
♦ Deciding what knowledge, skills and competencies are needed and developing a framework for these;
♦ Training, support and supervision amongst members of the team (including 6 monthly in-service training day);
♦ Multidisciplinary clinical audit and research;
♦ Journal club;
♦ Team charter and weekly caseload meetings.

The HEN team’s method of working in the community for routine patients’ review aids identification of when specialist knowledge and skills are needed. This will lead to a better use of staff resources. For example, more time will be made available for the SLT and dietitian to provide rehabilitation for patients. In addition, the NNS is able to deal with urgent requests such as tube blockages and displacements and provide training for nursing staff and carers to support these patients at home [34]. Through this multidisciplinary effort, there will be better patient outcomes and a more patient focused service as well as more opportunities for staff development, job satisfaction and better prospects for staff recruitment and retention.

However, to reach this stage takes time, building of trust between the different professions in the HEN team, and support for colleagues to take on new roles that are not normally associated with their traditional roles, e.g., SLTs rotating and advancing Percutaneous Endoscopic Gastrostomy (PEG) tubes [35,36]. Having regular training, away days, caseload review meetings and a team charter ensure that members of the HEN team have a sense of shared partnership. In addition, encouraging communication and supporting staff through change can lead to successful collaborative inter-disciplinary working. Extending roles may lead to greater job satisfaction and support staff retention. It would also enable better communication and information sharing and create opportunities for practice-based learning [9]. Role extension for nutrition nurses who become independent prescribers has the advantage of providing early and effective treatment for enteral feeding related complications without the need for doctor’s intervention. 

## 6. The Future of Enteral Feeding in the Community

**Figure 2:**
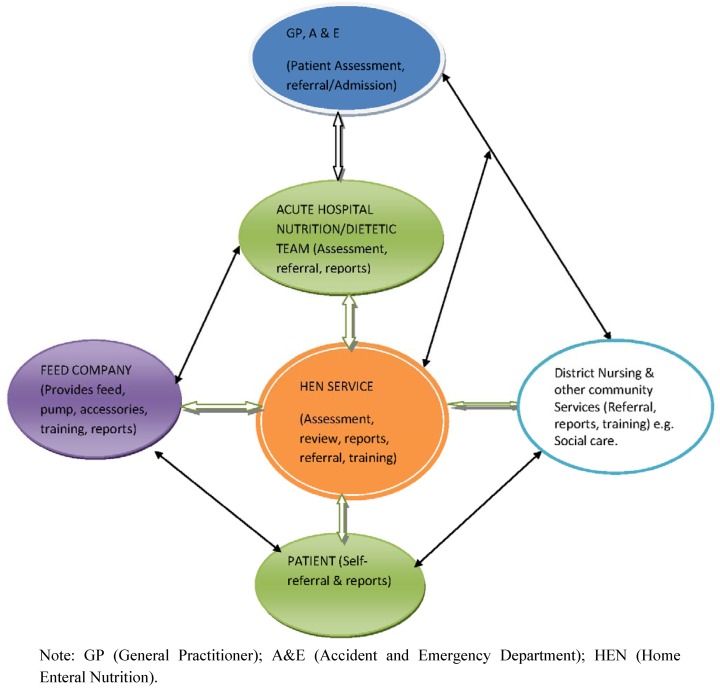
A model of the collaborative links between the HEN service, partner organizations and the patient.

The future of enteral tube feeding in the community relies on developing robust multidisciplinary HEN services with collaborative links with social care. A model of the HEN service with partnership links with external bodies is shown in Figure 2. The HEN service aims to provide specialist care, support and training for patients receiving home enteral nutrition. The coordination of enteral feed and equipment supplies and budget is also an essential part of the service. The skill mix in the HEN team has evolved in order to deliver an expert, comprehensive service to patients. While the different professional groups within the HEN team have distinct roles there are also similarities in their roles. The organizational structure of the HEN service is outlined in Figure 1.

The requirement to make extra time for patients and improve quality of care delivered as well as reduce costs means that “Service Leads” must ensure the safe and quality operational running of the clinical service for patients and that staff are well supported in terms of their professional and personal development. The staff need to understand how services will be funded in the future and they must be empowered to influence how the service is run and developed [37].

The HEN service should be developing clinical as well as patient reported outcomes to complement the data on patient activity, as increasingly evidence is needed to justify how services are set up and to secure funding and sustainability [7].

The NNS should be aiming to ensure that patients on HETF have adequate nutrition and hydration, are infection free and are independent with tube, stoma and pump care. Enteral tube feeding is often commenced in hospital setting and continues into the community especially in patients on long term tube feeding [38]. Although there are similarities in the dietetic roles in the hospital and community settings, it is essential to recognize that differences may be present in terms of access to advanced equipment such as those for biochemical and body composition measurements.

The HEN service hosted by Lewisham Healthcare is in a unique situation because it does not require the use of FP10 to prescribe enteral feed for the patients on enteral feeding based on the service level agreement with the feed-company [16]. However, patients that are being discharged home on enteral feeding usually have enough supply of feed, feeding pump, drip stand and giving sets that will last for about 5–7 days. This period will enable the HEN service take control of subsequent supplies and delivery of these items to the patient. This appears to be the future approach for enteral feeding in the community. 

Providing a comprehensive HEN service requires seamless patient care pathways between acute and community care settings [39]. A clear referral mechanism and good communication between healthcare professionals is essential [14]. Pivotal to success is the need for close links with referring acute units, in particular the NNS (where present), dietetic and SLT departments. As there is no adult NNS in some local acute units there are opportunities for HEN NNS to link with dietetic departments and ward staff to promote best practice in enteral tube feeding. This should lead to consistency in advice and training for patients and carers and fewer hospital admissions and clinical incidents [35].

Other partnerships may develop through the enteral feeding supplies contract with the enteral feed supplies and delivery company [16]. This process which involves tendering, monitoring and evaluation of the feed contract ensures that the HEN team provides patients a safe and quality service. The contract is monitored through a contract service agreement with key performance indicators, regular contract review meetings and teleconferences [16].

The contract may include company nurses who are integrated into the clinical team, which ensures good communication and consistency in care and advice and hopefully patients see a co-ordinated team and have a clear idea how to contact the team when needed, especially in an emergency [36].

The future of HEN service will be required to take on more roles and responsibilities including advocacy for people with learning disabilities.

## 7. Conclusion

There have been significant changes in health and social care provisions in the UK with and overarching impact on enteral feeding in the community. The factors driving the change include advances in technology, global economic recession and the increasing number of patients on enteral feeding in the community. Governments’ response such as the drive to move healthcare provisions from acute to community settings, develop NICE guidelines, integrate health and social care, enact the health and social Act are aimed at reducing costs, enhancing quality and promoting good clinical outcomes for patients on HETF. In this regard, new ways of working involving multidisciplinary teams such as the HEN team have been developed. This provides opportunity for cooperation and collaboration between different disciplines of healthcare professionals. However, this approach brings with it challenges and prospects for patients, professionals and providers within this area of practice. There have been extended roles for staff with the potential for increased clinical outcomes for patients. Challenges have also been evident in terms of the internal dynamics of the HEN service and the changing face of the health care delivery system at all levels of governance. 

## 8. Recommendations

In order to meet the future challenges of enteral feeding in the community brought on by changes in health and social care, it will be useful to:

♦ Develop a national framework for HEN service including the establishment of effective HEN team in all Community Services;
♦ Provide training opportunities for healthcare professionals (HCPs) working in the area of enteral feeding in the community and ensure that the funding arrangements for training is incorporated into service level agreements;
♦ Ensure adequate training of patients by specialist HCPs so that the patients can be independent in managing their own enteral feeding in the community;
♦ Extend the roles of HCPs including increasing the number of independent prescribers amongst HCPs who should be able to prescribe medicines, feeds and enteral nutrition supplements and accessories.
